# Benefits, Recruitment, Dropout, and Acceptability of the Strength Back Digital Health Intervention for Patients Undergoing Spinal Surgery: Nonrandomized, Qualitative, and Quantitative Pilot Feasibility Study

**DOI:** 10.2196/54600

**Published:** 2024-02-07

**Authors:** Annemieke van der Horst, Laura Meijer, Harmieke van Os - Medendorp, Jan S Jukema, Ernst Bohlmeijer, Karlein MG Schreurs, Saskia Kelders

**Affiliations:** 1 Research Group Smart Health Saxion University of Applied Sciences Deventer Netherlands; 2 Department of Psychology, Health and Technology Centre for eHealth & Well-being Research - Behavioural, Management and Social Sciences University of Twente Enschede Netherlands; 3 Roessingh Research & Development Enschede Netherlands; 4 Optentia Research Focus Area North-West University Vanderbijlpark South Africa

**Keywords:** pilot feasibility study, spinal surgery, digital health intervention, positive psychology, acceptance and commitment therapy, mobile phone

## Abstract

**Background:**

Patients undergoing spinal surgery report high levels of insecurity, pain, stress, and anxiety before and after surgery. Unfortunately, there is no guarantee that surgery will resolve all issues; postsurgical recovery often entails moderate to severe postoperative pain, and some patients undergoing spinal surgery do not experience (long-term) pain relief after surgery. Therefore, focusing on sustainable coping skills and resilience is crucial for these patients. A digital health intervention based on acceptance and commitment therapy (ACT) and positive psychology (PP) was developed to enhance psychological flexibility and well-being and reduce postsurgical pain.

**Objective:**

The objective of this study was 3-fold: to explore the *potential benefits* for patients undergoing spinal surgery of the digital ACT and PP intervention *Strength Back* (research question [RQ] 1), explore the feasibility of a future randomized controlled trial in terms of *recruitment and dropout* (RQ 2), and assess the *acceptability* of *Strength Back* by patients undergoing spinal surgery (RQ 3).

**Methods:**

We used a nonrandomized experimental design with an intervention group (n=17) and a control group (n=20). To explore the potential benefits of the intervention, participants in both groups filled out questionnaires before and after surgery. These questionnaires included measurements of pain intensity (Numeric Pain Rating Scale), pain interference (Multidimensional Pain Inventory), anxiety and depression (Hospital Anxiety and Depression Scale), valued living (Engaged Living Scale), psychological flexibility (Psychological Inflexibility in Pain Scale), and mental well-being (Mental Health Continuum–Short Form). Semistructured interviews combined with log data and scores on the Twente Engagement With eHealth Technologies Scale were used to assess the acceptability of the intervention.

**Results:**

A significant improvement over time in emotional (*V*=99; *P*=.03) and overall (*V*=55; *P*=.004) well-being (Mental Health Continuum–Short Form) was observed only in the intervention group. In addition, the intervention group showed a significantly larger decline in pain intensity (Numeric Pain Rating Scale) than did the control group (*U*=75; *P*=.003). Of the available weekly modules on average 80% (12/15) was completed by patients undergoing spinal fusion and 67% (6/9) was completed by patients undergoing decompression surgery. A total of 68% (17/25) of the participants used the intervention until the final interview. Most participants (15/17, 88%) in the intervention group would recommend the intervention to future patients.

**Conclusions:**

This pilot feasibility study showed that combining ACT and PP in a digital health intervention is promising for patients undergoing spinal surgery as the content was accepted by most of the participants and (larger) improvements in pain intensity and well-being were observed in the intervention group. A digital intervention for patients undergoing (spinal) surgery can use teachable moments, when patients are open to learning more about the surgery and rehabilitation afterward. A larger randomized controlled trial is now warranted.

## Introduction

### Background

Patients undergoing spinal surgery report high levels of physical complaints as well as insecurity, pain, low well-being, stress, and anxiety before and after surgery [1-3]. Postsurgical recovery often entails moderate to severe postoperative pain, and approximately 20% to 30% of patients undergoing spinal surgery do not experience (long-term) pain relief after surgery [3,4]. This results in a longer hospital stay, higher health care costs, longer physical and mental recovery, delayed return to work, and the potential development of chronic pain. The potential transition from postoperative pain to chronic pain is a major issue as chronic pain affects many aspects of a patient’s life, such as work; physical, emotional, and social well-being; and quality of life [5,6].

Mental factors such as cognition, emotions, and expectations play a significant role in the experience of pain [7]. The fear-avoidance model explains the trajectory from acute to chronic pain through fear and catastrophizing, which is the tendency to enlarge the threat of pain and a feeling of helplessness, leading to an increase in pain avoidance as a dominant coping strategy [8]. Pain avoidance then leads to a less active lifestyle, thereby worsening rather than relieving pain. High levels of catastrophizing and fear have been found to predict higher levels of (postoperative) pain and pain chronicity and a lower quality of life in patients who undergo surgery [4,9-11]. In addition, realistic expectation management is key as unrealistic or unfulfilled expectations about surgery and preoperative stress may lead to the experience of higher levels of postoperative pain [12-18]. This link between mental factors and expectation management with postoperative outcomes shows that psychological preparation before surgery is essential to improve recovery after surgery.

Although the potential benefits of psychological preparation before surgery have long been known [19], a more recent meta-analysis of psychological preparation techniques before surgery could not find strong evidence from high-quality research to verify these claims [20]. Potentially, interventions that focus on promoting adaptive coping skills and reducing maladaptive emotion regulation skills are more promising. Smith and Zautra [21] and Sturgeon and Zautra [22] found, for example, evidence that coping strategies such as (pain) acceptance, engaging in beneficial social interactions, and experiencing a value-based purpose in life have the potential to improve mental well-being and promote resilience in the face of (chronic) pain. Sustainable resilience to (chronic) pain requires skills promoting adaptation and mental health in the long term [22]. In other words, adaptation to different contexts and circumstances requires (psychological) flexibility and a long-term focus. In the context of pain, psychological flexibility implies that painful sensations, feelings, and thoughts are accepted as opposed to avoided and that attention shifts toward personally valued goals [23]. For patients undergoing spinal surgery, psychological flexibility enables them to cope with the fluctuating circumstances surrounding surgery (eg, insecurity, fear, and pain) in a flexible rather than rigid manner. Being able to accept negative emotions or sensations such as pain and insecurity in the face of surgery might prevent catastrophizing, fear, and avoidance behavior.

Psychological flexibility is the aim of acceptance and commitment therapy (ACT) [24]. ACT is based on the relational frame theory and focuses on performing value-based activities in life even in the face of insecurity and adversity [24]. In a similar way, positive psychology (PP) is the scientific study of well-being and optimal functioning, focusing on human flourishing instead of reducing the risk factors for psychopathology and malfunctioning. PP involves topics such as strengths, virtues, meaning, happiness, gratitude, compassion, resilience, and flourishing [25]. ACT and PP can help experience a value-based purpose in life, promote resilience, and improve mental well-being. Mental well-being comprises positive emotional, psychological, and social functioning [26]. The presence of higher levels of these 3 dimensions of well-being is an indicator of flourishing [26-28]. Promoting positive resources and skills that contribute to successful adaptation and (mental) health are the aims of PP interventions (PPIs) [25,29]. PPIs and ACT have been found to be effective in the treatment of chronic pain [30-35], improving affect and functional ability after knee surgery [21], and quicker cessation of pain and opioid use in veterans after orthopedic surgery [36]. In summary, PP and ACT could potentially benefit patients undergoing spinal surgery in terms of physical (eg, pain) and psychological (eg, well-being) factors.

In the context of surgery, digital health interventions can be used to aid patients during their recovery process [37]. These interventions can be used to improve postoperative outcomes by supporting healthy lifestyle behavior change before and after surgery [38,39] and to improve medication adherence [40,41]. Digital health interventions can also better prepare patients for surgery or shorten postoperative recovery through behavioral modification, patient monitoring, or protocol adherence [42-45]. Behavioral modification can also be applied to promote a healthy lifestyle. This is important as healthy lifestyle behavior changes before and after orthopedic surgery, such as increased preoperative physical activity or smoking cessation, have been associated with improved postoperative bone healing [46] and wound healing [47], quicker recovery times, and reduced pain scores [48]. Moreover, patients undergoing surgery who engage with digital health interventions show better medication adherence, better adherence to discharge instructions, greater patient satisfaction, improved clinic attendance, lower readmission, and less emergency department visits after surgery [49]. van der Meij et al [50] found in their review that, in most studies, perioperative digital health interventions improved clinical patient-related outcomes compared with face-to-face perioperative care alone for patients who had undergone various forms of surgery. This shows the potential of digital health interventions to improve perioperative care in addition to face-to-face meetings with professionals.

Despite the clear potential of ACT and PPIs for mental well-being and long-term resilience, they have not yet been used to develop interventions targeting psychological flexibility in patients undergoing spinal surgery. In addition, seeing the potential of digital health interventions in the context of surgery, this form also seems promising for patients undergoing spinal surgery. For this reason, a digital health intervention called *Strength Back* [51] was developed. *Strength Back* aims to increase psychological flexibility and well-being and improve postoperative recovery in patients undergoing spinal surgery. This intervention, developed through cocreation with different stakeholders, contains procedural information, pain education, and PPI and ACT exercises.

### Objectives

Although the intervention is based on a scientific, theoretical framework, the effects and impact of the intervention need to be proven to be able to implement it in an evidence-based health care setting. This requires a collection of evidence, such as through a randomized controlled trial (RCT). As an RCT is complex and expensive, preparation and insights in advance into the required parameters, such as measures, recruitment, and dropout numbers, are essential to perform a thorough RCT. In light of the aforementioned research on PP and ACT in other target populations, several outcome measures covering physical and psychological factors need to be explored as potential benefits of this intervention. In addition, in an RCT, the best possible version of the intervention needs to be tested. This is in line with the recommendation of the Medical Research Council of first testing and refining an intervention to ensure it is acceptable to successfully evaluate whether it is effective [52]. In addition, to reduce psychological and practical barriers and maximize acceptability, it is important to understand and accommodate patients’ views [53]. As of yet, it is unknown how patients undergoing spinal surgery value the intervention *Strength Back* in terms of acceptability, potential benefits, and feasibility in a real-life setting and what the required parameters for a future RCT would be. Therefore, the objective of this study was 3-fold: to explore the *potential benefits* for patients undergoing spinal surgery of the digital ACT and PPI *Strength Back* (research question [RQ] 1), explore the feasibility of a future RCT in terms of *recruitment and dropout* (RQ 2), and assess the *acceptability* of *Strength Back* by patients undergoing spinal surgery (RQ 3).

## Methods

### Study Design

**Figure 1:**
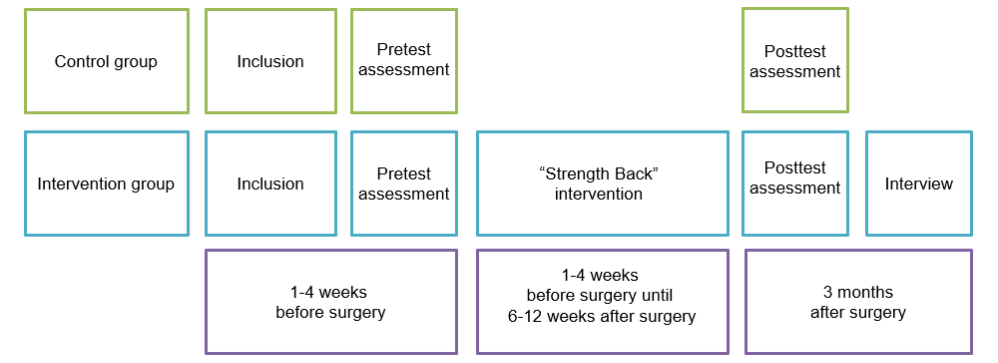
Study design and timeline for individual participants (patients undergoing spinal surgery) in the control and intervention groups of this pilot feasibility study.

This was a nonrandomized pilot study, a subset of feasibility studies as described by Eldridge et al [54]. Our study design included an intervention group and a historic control group (Figure 1). We used a historic control group as the development of the intervention was still ongoing and the target population was small. The participants in the control group received care as usual. In addition to receiving care as usual, the participants in the intervention group were given access to the digital health intervention *Strength Back*. To test the acceptability of the intervention, individual interviews were conducted with the participants in the intervention group. Both groups filled out a questionnaire before (pretest time point) and after (posttest time point) surgery.

As suggested by Orsmond and Cohn [55], we used a combination of qualitative and quantitative measures in this feasibility study. We aimed to include 20 participants in our control group and 20 participants in our intervention group, which is in line with the study by Billingham et al [56], who found a median sample size of 36 in their review of feasibility studies.

### Participants

Patients undergoing decompression or spinal fusion surgery were eligible for inclusion in either the control or intervention group of this study. Patients undergoing spinal surgery other than decompression or spinal fusion surgery (eg, patients with hernias or patients of oncology) were excluded. Other inclusion criteria were age of >18 years, proficiency in Dutch, and an email address. Participants in the intervention group needed an Android or iOS smartphone or tablet at home and had to be willing to use a digital health intervention both before and after surgery.

All participants in this study were recruited through purposive sampling at an orthopedic center in the Netherlands. The participants in the historic control group were recruited between February 2020 and July 2020. The participants in the intervention group were recruited between September 2020 and February 2021.

### Conditions

#### Care as Usual

The historic control group received care as usual. They were provided with brochures containing information about their diagnosis, surgical procedure, surgery preparation, physical guidelines during recovery, contact information, and possible complications. In addition, usual care consisted of several appointments besides the surgery itself: an intake and a preoperative screening before surgery, a consultation by phone 10 days after surgery, and a physical checkup at the orthopedic center 6 weeks (both surgery types) and 12 weeks (spinal fusion surgery) after surgery.

#### Strength Back Digital Health Intervention

**Figure 2:**
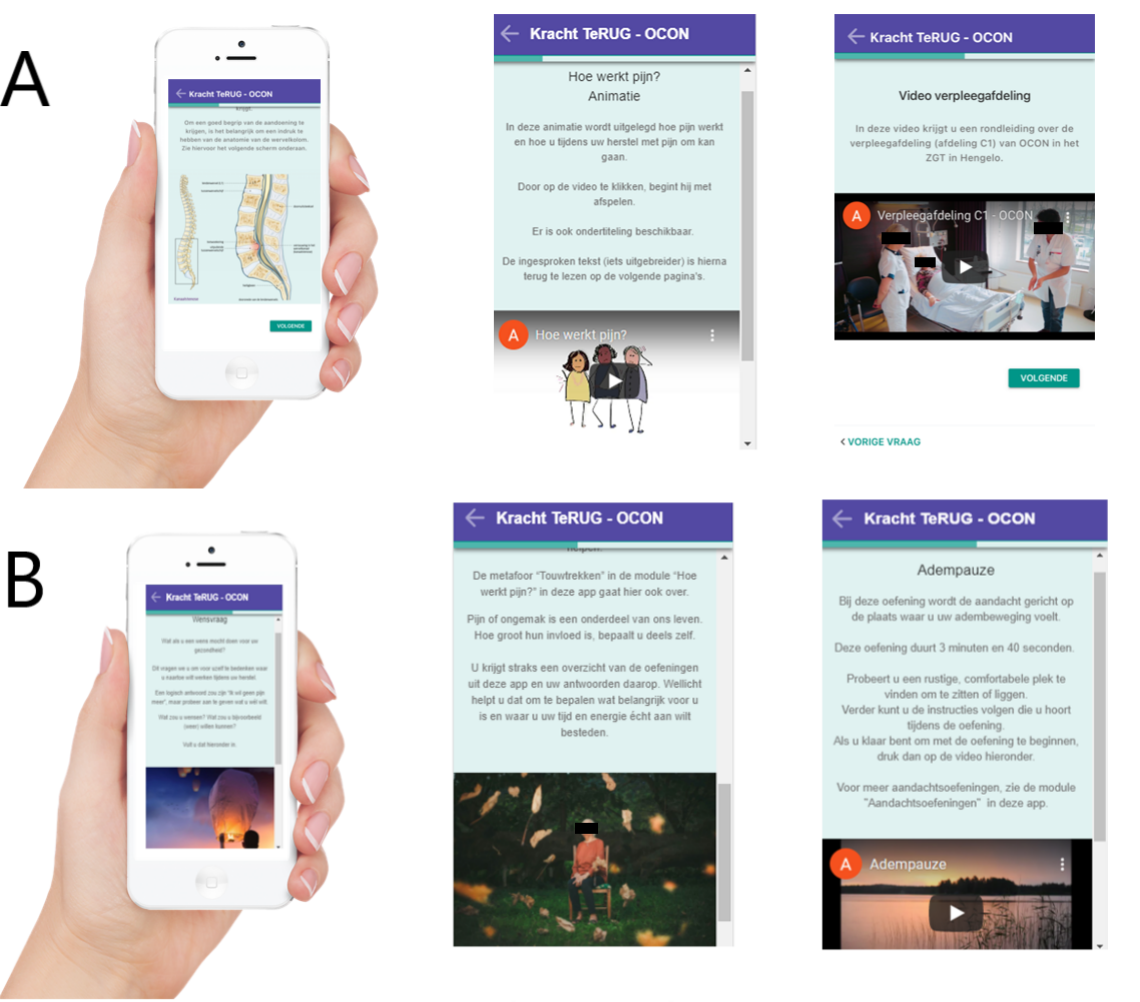
Screenshots of the Strength Back intervention (in Dutch, Kracht TeRUG) for patients undergoing spinal surgery. (A) The information on the spinal condition, the animation on pain education, and the video of the nursing ward. (B) The “wish question,” text about acceptance of pain, and “mindful breathing” exercise.

In addition to care as usual, participants in the intervention group were given access to the *Strength Back* digital health intervention (see Figure 2 for screenshots). The content of this app was developed through a process of cocreation. The stakeholders involved in this process were patients who had undergone spinal surgery, orthopedic surgeons, a physical therapist, a nurse practitioner, a research coordinator, and several nurses. The content of the intervention was based on the results of interviews and focus group sessions with the aforementioned stakeholders (patients and health care professionals) combined with ACT and PP exercises. Multimedia Appendices 1 to 3 of this publication and our previous publication on the cocreation of *Strength Back* [51] provide more details on the developmental process.

The intervention consisted of 13 information modules, which were continuously available, and 6 to 12 weekly modules. Participants did not receive personal messages from a health care professional. The contact information of the orthopedic clinic was provided in the intervention, making sure participants could contact a health care professional in case of any questions.

The information modules of the intervention were based on existing brochures and leaflets of the orthopedic center (Multimedia Appendix 1).

In addition, to support them during recovery, intervention participants received 6 to 12 weekly modules. These modules were timed: participants received 3 modules in the 4 weeks before surgery and 6 or 12 weekly modules after surgery depending on the type of surgery they had undergone. In total, 6 weekly modules were provided for patients undergoing decompression surgery, and 12 weekly modules were provided for patients undergoing spinal fusion, matching the expected recovery time after surgery as suggested by the orthopedic center (Multimedia Appendices 2 and 3).

During the intervention, participants received automated reminders every time a new weekly module was available. This was voluntary and could be turned on or off by the participants.

The digital health intervention was downloaded by the participants themselves using a step-by-step guide provided by the researcher.

### Procedure of Data Collection

For both conditions, patients received a leaflet from their personal health care professional (ie, orthopedic surgeon, specialized physical therapist, or advanced nurse practitioner) explaining this study during their visit to the orthopedic center and were subsequently contacted by phone by the researcher. When patients agreed to take part in the study, their email address was collected, after which they received an information email with a link to the first web-based questionnaire (through Qualtrics [Qualtrics International Inc]; pretest assessment). We chose this specific moment for the pretest assessment to clarify the current and most up-to-date picture of the preoperative situation. In the questionnaire, the participants first had to provide active informed consent to continue. Participants in both the control and intervention groups filled out this questionnaire (pretest assessment; Figure 1).

A total of 3 months after surgery, participants in both groups received an invitation by email to fill in the posttest questionnaire. At this time, it was expected that (nearly) all participants were in the final phase of their recovery process. We chose this specific moment to collect the data, when the recovery situation had stabilized. At this point, the participants in the intervention group were contacted by the principal investigator (AH) to make an appointment for an interview to assess how they valued the intervention. These semistructured interviews were conducted between January 2021 and June 2021 by 2 researchers (AH and LM). Owing to the COVID-19 pandemic, interviews were conducted via telephone. The interviews were audio recorded and lasted between 30 and 60 minutes.

The recruitment and dropout numbers in the intervention group were explored in this study to provide insights into what is needed for the recruitment process of a future RCT to reach the desired power.

### Materials

Regarding RQ 1 (potential benefits), the questionnaires contained several outcome measures (ie, pain intensity and interference, anxiety, depression, psychological inflexibility, valued living, and mental well-being). These potential benefits were measured at the pre- and posttest time points for both the control and intervention groups.

Pain intensity was measured using the Numeric Pain Rating Scale (NRS). For the NRS, participants were asked to circle the number (0-10) that best described their experience of pain in the last week in general and in the last week at worst. The questions asked were as follows: “Please indicate the average intensity of your pain in the last week” and “Please indicate the intensity of your pain at the worst moments in the last week.” A higher score indicates a higher intensity of pain. The NRS is considered a valid, reliable, and appropriate pain rating scale for use in clinical practice, with good sensitivity [57].

Pain interference was measured using the pain interference subscale of the Multidimensional Pain Inventory (MPI) [58]. This subscale consists of 11 items measuring to what degree pain influences the daily life of a respondent, with total mean scores ranging from 0 to 6. A higher score indicates a higher degree of pain interference. The subscale showed good reliability in our study population, with a Cronbach α of .874.

Anxiety and depression were measured using the Hospital Anxiety and Depression Scale (HADS) [59]. Both the anxiety and depression subscales consist of 7 items with scores ranging from 0 to 21. A higher score indicates a higher degree of anxiety or depression. Both subscales showed good reliability, with a Cronbach α of .874 for the anxiety subscale and .789 for the depression subscale.

Psychological inflexibility was measured using the Psychological Inflexibility in Pain Scale (PIPS) [60]. The 2 subscales measure avoidance and cognitive fusion, concepts derived from the theoretical framework of ACT [24]. As the cognitive fusion subscale showed low reliability in our study population with a Cronbach α of .524, only the total score of the PIPS was used in this study. This total score had good reliability, with a Cronbach α of .893. A higher score indicates more inflexibility with pain (ie, lower acceptance and higher cognitive fusion).

Value-based living was measured using the “Valued living” subscale of the Engaged Living Scale (ELS; Trompetter et al [61]), which covers the concept of “valued living”—recognizing personal values and undertaking committed actions that are congruent with these values. The “Valued living” subscale consists of 10 items and uses a 5-point scale, with scores ranging from 10 to 50. A higher score indicates a higher degree of valued living. This subscale of the ELS showed high reliability, with a Cronbach α of .917.

Mental well-being was measured using the Mental Health Continuum–Short Form [62] on three dimensions: (1) emotional well-being, defined as positive feelings (3 items); (2) psychological well-being, described as individual functioning (eg, environmental mastery and purpose in life; 6 items); and (3) social well-being, measuring functioning in community life (eg, social contribution and social acceptance; 5 items). The mean scores per subscale range from 0 to 5, with a higher score indicating a higher degree of emotional, psychological, or social well-being. The total score (Cronbach α=.930) as well as that of the different subscales (emotional: Cronbach α=.930; psychological: Cronbach α=.874; social: Cronbach α=.799) showed good reliability, with a high Cronbach α in our study population.

Regarding RQ 2 (recruitment and dropout), we kept an overview in Microsoft Excel (Microsoft Corp) of the recruitment and dropout numbers in the intervention group in this study.

Regarding RQ 3 (acceptability), the scheme used for the interviews consisted of 2 parts. The first part of the interview started with questions on the intervention in general (eg, topics on layout and user-friendliness based on the Mobile App Rating Scale [63]). The second part continued by discussing the content of the modules (Multimedia Appendix 1) followed by the exercises in the weekly modules (Multimedia Appendix 2) as topics. Engagement with the intervention was measured using the Twente Engagement With eHealth Technologies Scale (TWEETS) [64]. This scale of 9 items comprises 3 subscales: behavioral, cognitive, and affective engagement. In this study, the total score, with a Cronbach α of .883, was used. In addition, the automated logs from the system were analyzed to see when participants opened which weekly module. The information modules were continuously available (before and after surgery), and activity in these modules was not recorded in the automated logs.

### Data Analysis

All statistical analyses were conducted using SPSS (version 26.0; IBM Corp). For the exploratory aim of this study, full cases provide the most valuable information. Therefore, only full-case analyses were conducted. In addition, as the quantitative data were used to generate a general picture of between-group differences, no imputation techniques were used. When participants filled out the pre- or posttest assessment more than once, the first completed test was used for the analysis.

For RQ 1 (potential benefits), between-group differences were assessed using nonparametric Mann-Whitney *U* tests because of the small sample size and nonnormal distribution of the data. First, between-group differences were analyzed at baseline using the Mann-Whitney *U* test. Second, within-group differences were analyzed for both groups between the pre- and posttest assessment using a Wilcoxon signed rank test. The effect sizes were calculated using the Cohen *d*: (mean at the posttest assessment – mean at the pretest assessment) / (SD). When the SD of both moments differed, the mean SD of the pre- and posttest assessments was calculated and used. Values for the Cohen *d* of 0.2 were considered a small effect size, 0.5 was considered a medium effect size, and values of ≥0.8 were considered a large effect size.

Third, between-group differences were analyzed by comparing the pre- and posttest difference scores of both groups (Mann-Whitney *U* test). All statistical tests were 2 sided. *P* values of <.05 were considered statistically significant. As this was a feasibility pilot study, the results were considered exploratory, and no correction for multiple testing was performed.

For RQ 2 (recruitment and dropout), we analyzed the recruitment and dropout numbers of the intervention group in this study. For the analysis, we looked at the number of approached patients, the number of patients who consented to participate in the study, and the time span in which all these patients were approached. Dropout was defined as agreeing to participate in the study but not downloading the intervention, not filling out the pretest questionnaire, or not filling out the posttest questionnaire.

For RQ 3 (acceptability), interview data were collected, and the audio recordings were transcribed verbatim. For the analysis, the transcripts of all interviews were read and reread by 2 researchers (AH and LM) to familiarize themselves with the data. Subsequently, the transcripts were open coded by both researchers independently, followed by a discussion to reach a consensus regarding the coding. As a next step, both researchers sorted the codes into several groups. A deductive coding approach was used based on the topics on the interview scheme combined with an inductive approach for important content within these topics. For the coding and analysis process, the ATLAS.ti software (version 9.0; ATLAS.ti Scientific Software Development GmbH) was used.

### Ethical Considerations

Ethics approval was granted by the Medical Ethical Committee of the Radboud University Medical Centre in Nijmegen (2019-5608) and the Ethics Committee of the Faculty of Behavioural, Management, and Social Sciences of the University of Twente (191080). The study is registered at the repository of the University of Twente for processing of personal data (AVG Register; WBP18ME0048).

Participants in both the control group and the intervention group filled out a questionnaire before and after surgery. When patients agreed to take part in the study, they received a link to the first questionnaire, in which they had to provide active informed consent to continue.

All human participant data were deidentified before analysis. Study participants received no compensation for their inclusion in the study.

## Results

### Sample

**Figure 3:**
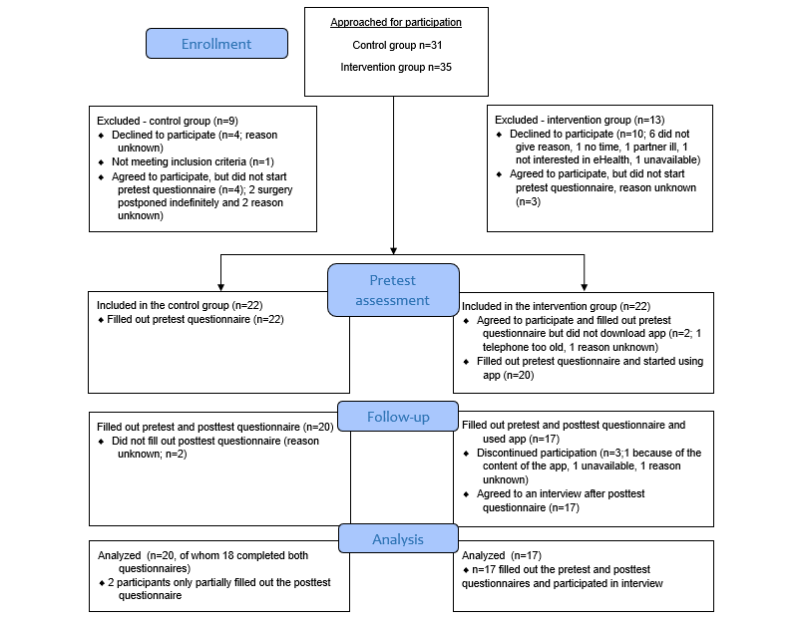
Flowchart of participants (patients undergoing spinal surgery) in the intervention and control groups of this pilot feasibility study.

Figure 3 shows an overview of the inclusion of participants in the intervention and control groups. A total of 25 patients agreed to participate in the intervention group, of whom 3 (12%) participants did not fill out the pretest questionnaire; the reason for this is unknown. Therefore, a total of 22 participants filled out the pretest questionnaire. After filling out the pretest questionnaire, 9% (2/22) of the participants failed to download the intervention to their phones. Therefore, 20 participants started using the intervention (20/35, 57% of the approached patients). Before the posttest questionnaire, 15% (3/20) of the patients discontinued their participation. This resulted in a total of 17 participants filling out the pre- and posttest questionnaires. All these participants (17/17, 100%) also took part in a telephone interview.

A total of 31 patients were approached by phone to participate in the control group. This resulted in 71% (22/31) of these participants completing the pretest questionnaire. A total of 9% (2/22) of these participants did not complete the posttest questionnaire without giving a reason. In total, 9% (2/22) of the participants started the posttest questionnaire but only partially completed it. This resulted in a control group of 20 participants, of whom 18 (90%) completed the entire pre- and posttest questionnaires.

The participant characteristics at baseline are presented in Table 1. The Mann-Whitney *U* test revealed no substantial differences between the intervention and control groups in terms of these characteristics.

**Table 1:** Participant characteristics at baseline by group.

		Intervention group (n=17)	Control group (n=20)
**Sex, n (%)**			
	Female	6 (35)	12 (60)
	Male	11 (65)	8 (40)
Age (years), mean (SD)		62 (12)	66 (14)
**Surgery type, n, (%)**			
	Decompression	11 (65)	10 (50)
	Spinal fusion	6 (35)	10 (50)
**Educational level^a^, n (%)**			
	Low	4 (24)	9 (45)
	Middle	8 (47)	4 (20)
	High	5 (29)	7 (35)
**Occupational status, n (%)**			
	Paid work	6 (35)	8 (40)
	Retired	8 (47)	10 (50)
	Housewife or househusband	1 (6)	2 (10)
	Incapacitated	1 (6)	0 (0)
	Self-employed	1 (6)	0 (0)
**Marital status, n (%)**			
	Married or living together	15 (88)	18 (90)
	In a relationship; living apart	1 (6)	0 (0)
	Widowed	1 (6)	2 (10)

^a^Low: primary and lower secondary education; middle: upper secondary education; high: higher vocational training and university.

### RQ 1: What Are the Potential Benefits for Patients Undergoing Spinal Surgery of a Digital ACT and PPI?

First, between-group differences were analyzed at baseline (Mann-Whitney *U* test). The total score on the PIPS was significantly higher in the control group (*U*=262.5; *P*=.004). Other measures did not differ significantly between both groups at baseline.

Second, within-group differences were analyzed for both groups between the pre- and posttest assessments (Wilcoxon singed rank test; see Table 2 for the results). When looking at the differences between the pre- and posttest assessment, it should be taken into account that both groups had undergone surgery, which is an intervention by itself. Both groups showed significant improvement in the NRS (pain intensity week average and week worst), HADS anxiety subscale, HADS depression subscale, pain interference subscale of the MPI, and total score on the PIPS between the pre- and posttest time points. The score on the valued living scale of the ELS did not significantly change over time in either of the 2 groups. In the control group, the subscores as well as the total score on the Mental Health Continuum–Short Form did not differ significantly between the pre- and posttest time points. In the intervention group, the total score (*V*=99; *P*=.03 as well as the score on emotional well-being (*V*=55; *P*=.004) increased significantly over time.

**Table 2:** Potential benefits for patients undergoing spinal surgery in the control and intervention groups.

		Pretest assessment, mean (SD)	Posttest assessment, mean (SD)	Pretest-posttest difference, *V*	Pretest-posttest difference, *P* value	Pretest-posttest effect size, Cohen *d*	Pretest-posttest mean difference—full sample (95% CI)	
**PIPS^a^—total**								−13.34 (−19.19 to −7.50)
	Control group (n=18)	62.2 (9.8)	49.7 (11.2)	19	.004	1.19		
	Intervention group (n=17)	52.5 (11.6)	43.8 (11.7)	24.5	.01	0.75		
**NRS^b^—week average**								−3.80 (−4.57 to −3.03)
	Control group (n=20)	6.7 (2.3)	3.8 (2.6)	0	<.001	1.18		
	Intervention group (n=17)	7.1 (1.1)	2.0 (1.6)	0	<.001	3.71		
**NRS—week at worst**								−4.09 (−4.94 to −3.23)
	Control group (n=20)	8.5 (1.5)	5.0 (3.1)	0	<.001	1.44		
	Intervention group (n=17)	8.3 (1.2)	3.3 (2.5)	0	<.001	2.55		
**HADS^c^—anxiety**								−1.89 (−2.92 to −0.86)
	Control group (n=20)	7.3 (3.8)	5.2 (3.8)	14	.008	0.55		
	Intervention group (n=17)	4.6 (3.2)	2.8 (2.2)	13.5	.02	0.66		
**HADS—depression**								−3.46 (−4.62 to −2.29)
	Control group (n=20)	7.0 (3.8)	4.3 (3.4)	26.5	.006	0.75		
	Intervention group (n=17)	5.8 (3.3)	1.9 (1.8)	4.5	<.001	1.47		
**ELS^d^—valued living**								−0.60 (−2.28 to 1.08)
	Control group (n=18)	37.8 (4.9)	36.5 (5.9)	50	.57	0.24		
	Intervention group (n=17)	40.4 (5.5)	40.2 (5.8)	57.5	.89	0.04		
**MHC-SF^e^—emotional**								0.46 (0.12 to 0.80)
	Control group (n=18)	3.4 (1.2)	3.8 (0.7)	73.5	.44	0.41		
	Intervention group (n=17)	3.7 (1.2)	4.2 (0.7)	55	.004	0.51		
**MHC-SF—social**								0.12 (−0.14 to 0.38)
	Control group (n=18)	2.7 (1.1)	2.7 (1.1)	89	.55	0		
	Intervention group (n=17)	2.8 (1.0)	2.9 (1.2)	63	.21	0.09		
**MHC-SF—psychological**								0.21 (−0.14 to 0.57)
	Control group (n=18)	3.1 (1.1)	3.3 (1.0)	86	.35	0.19		
	Intervention group (n=17)	3.6 (1.0)	3.7 (1.0)	80	.08	0.10		
**MHC-SF total**								0.23 (−0.04 to 0.50)
	Control group (n=18)	3.0 (1.1)	3.2 (0.9)	106.5	.36	0.19		
	Intervention group (n=17)	3.3 (0.9)	3.5 (0.9)	99	.03	0.22		
**MPI^f^—pain Interference scale**								−2.07 (−2.59 to −1.55)
	Control group (n=19)	4.4 (0.9)	2.6 (1.4)	9.5	<.001	1.53		
	Intervention group (n=17)	4.1 (0.9)	1.6 (1.1)	0	<.001	2.49		

^a^PIPS: Psychological Inflexibility in Pain Scale.

^b^NRS: Numeric Pain Rating Scale.

^c^HADS: Hospital Anxiety and Depression Scale.

^d^ELS: Engaged Living Scale.

^e^MHC-SF: Mental Health Continuum–Short Form.

^f^MPI: Multidimensional Pain Inventory.

Third, between-group differences were analyzed by comparing the pre- and posttest difference scores of both groups (Mann-Whitney *U* test). A significant difference was found in the weekly average NRS score between the intervention and control groups (*U*=75; *P*=.003), showing a significantly larger decline in pain intensity in the intervention group.

### RQ 2: What Is the Feasibility of a Future RCT in Terms of Recruitment and Dropout Rates?

To determine the feasibility of a future RCT, recruitment, dropout rates, and dropout characteristics were examined. Over a period of 5 months, a total of 35 patients were approached by phone to participate in the intervention group (Figure 3). In total, 51% (18/35) of the approached patients did not want to participate and did not start with or dropped out of the intervention. Of these 18 patients, 14 (78%) were female and 14 (78%) underwent decompression surgery. Most of the dropouts did so before the intervention started as they did not want to participate in the study (10/18, 56%, of whom 9/10, 90% were female; age range 19-79 years). Most of the additional 44% (8/18) of dropouts did so at the pretest (3/8, 38%) or posttest (3/8, 38%) time points, and only 25% (2/8) of these participants dropped out because of nonadherence as they did fill out the pretest questionnaire but did not download the intervention. Of these 2 nonadherers, 1 (50%) was male and 1 (50%) was female; both underwent decompression surgery, and both were aged >65 years.

### RQ 3: What Is the Acceptability of a Digital ACT and PPI Called “Strength Back” by Patients Undergoing Spinal Surgery?

#### Overview

Individual semistructured interviews were conducted with all 17 participants in the intervention group. The results of these interviews are described in 3 sections: general impression of the intervention, content of the intervention, and suggestions for improvement. Subsequently, engagement with the intervention is discussed by combining input from the interviews, log data, and the scores on the TWEETS [64].

#### General Impression of the Intervention

The general impression of the intervention can be described in terms of *added value*, *recommendation*, *ease of use*, *repeated answers*, *feedback*, and *notifications*.

Most of the participants (15/17, 88%) felt that the intervention had at least some added value in terms of support and as a source of information. PT3 stated the following:

I always liked it when there was a new module that you had to read, I always found it very interesting. Because each time I read the app, it might contain something that really helps me. That helps me recover faster perhaps.

PT3

Even when some participants at first stated that they did not really think the intervention was useful, they corrected themselves later in the interviews. For instance, PT8 stated the following:

It did make me realize that the surgery was not a thing to take too lightly, that I could experience a relapse, so it did comfort me to know that in advance.

PT8

Of the 17 participants who were interviewed, 2 (12%; PT4 and PT5) stated that the intervention did not have any added value for them. For instance, PT5 stated the following:

The information from the physician was enough for me, but it might be helpful for other patients.

PT5

A total of 12% (2/17) of the participants (PT4 and PT8) stated that they found the intervention to be too fluffy or vague and not suitable for them as they did not experience any pain or ups and downs.

When comparing the intervention with the paper brochure that patients normally receive from the hospital, some participants stated that either one would have been fine (eg, PT1 and PT9), whereas others stated that they preferred the intervention as the information was always nearby and, therefore, more accessible than a paper brochure that might get lost (eg, PT2, PT5, and PT6). Some participants stated that they had looked on the internet for information as well but preferred the information in the intervention as this was tailored to their own hospital and, therefore, also more reliable.

Similarly, 88% (15/17) of the participants stated that they would recommend the intervention; one participant (PT4) would not recommend it as it did not offer him anything additional to a paper brochure, and with another participant (PT12), this topic was not discussed. Participants mainly stated that they would recommend the intervention as it helped them prepare for surgery and enabled them to read and reread important information.

Participants indicated that the intervention was easy to use. A total of 12% (2/17) of the participants (PT5 and PT18) mentioned some minor layout suggestions, but the main consensus was that the intervention was easy to download and use, had a nice layout, and contained text in clear language. The functionality of previous answers (eg, preoperative answers on valuable activities to do after surgery) being repeated in later modules was appreciated by the participants:

Nice to read what I had filled in previously and to see, on a later date that I was doing better when I answered a similar question.

PT3

As the intervention only provided general information and no personal feedback from a professional, participants were asked whether they had missed this. Several participants (5/17, 29%) indicated that they did miss this personal feedback during the intervention or in general during recovery. This preferred feedback ranged from the ability to click on a help button (PT15) or the possibility of contacting someone for more information (PT1) to reaching out to someone guiding the intervention to reduce the amount of psychological content (PT11 and PT16) or feeling insecure and missing a contact person to consult (PT7).

Owing to an error, participants did not correctly receive a notification every time a new weekly module was available. Participants (6/17, 35%) indicated that receiving a notification made them use the intervention more. They would have liked to receive the reminder every week, stating that they would have used the intervention more if they had.

#### Content of the Intervention

During the interview, all separate components of the intervention (Multimedia Appendices 1 and 2) were discussed with the participants. These elements were clustered as *preparation and peer support*, *hospital information*, and *positive psychology and ACT content*.

##### Preparation and Peer Support

Almost all participants (14/17, 82%) appreciated the videos of the nursing ward and the surgery room. These participants stated that they found watching the videos informative and comforting. One of the participants who did not appreciate the videos (PT11) stated the following:

It might be comforting for little children, but as an adult you know what a hospital looks like and what will happen.

PT11

The practical tips from previous patients were valued by most participants (11/17, 65%). In total, 24% (4/17) of the participants did not see or remember this element, and 12% (2/17) of the participants would have liked to see more tips. The module describing the fluctuating recovery process was not remembered by several participants (8/17, 47%) but was valued greatly by those who did remember it:

I could really recognize myself in the text, having had pain for so long myself.

PT14

It really resembled the reality.

PT18

Participants reacted with mixed feelings to the quotes of previous patients. In total, 12% (2/17) of the participants (PT1 and PT16) stated that it might benefit other people but it was not for them. Several participants (7/17, 41%) could not remember the module (PT2, PT8, and PT15) or did not find it interesting (PT4, PT9, PT11, and PT13). These participants stated that they wanted to do things their own way and were not interested in the stories of other patients. Other participants (8/17, 47%) found the quotes valuable, recognizable, and comforting.

##### Hospital Information

The information on the spinal condition and on the surgical procedure was appreciated by the participants. Some stated that it was clearer than what the physician had told them or that it was nice to be able to read and reread it in the intervention, whereas other participants stated that it had no added value to them as the information resembled that of the paper brochure. The information on physical therapy did not match reality for some participants (3/17, 18%), but the discharge criteria were useful to see before surgery. In total, 18% (3/17) of the participants (PT1, PT4, and PT9) thought that the physical guidelines in the intervention had no added value as they were also available in the paper brochure. Another 18% (3/17) of the participants (PT11, PT15, and PT18) liked the fact that the physical guidelines were in the intervention but would have preferred them to be more specific or elaborate. The other participants (11/17, 65%) found the guidelines pleasant, useful, and supportive. Most participants (11/17, 65%) appreciated the pain medication module. The other participants (6/17, 35%) preferred the paper brochure, could not remember the module, did not see the module, or did not use it. The contact details of the hospital in the intervention were considered clear and useful by all participants.

##### PP and ACT Content

The PP and ACT content in the intervention evoked the strongest opinions in the participants (Table 3). Several participants (5/17, 29%) stated that the amount of psychological content in the intervention was too high and made them less engaged with the intervention (like or use it less):

The amount of psychological content was too much for me. I wasn’t raised that way and it doesn’t appeal to me. It almost made me stop using the intervention all together.

PT17

**Table 3:** Participants’ opinions on the positive psychology and acceptance and commitment therapy content of the intervention ranked in order of most to least appreciated exercise (n=17).

Exercise	Appreciated, n (%)	Not appreciated, n (%)	Not remembered, n (%)	Quote
Emotion quadrant	13 (76)	4 (24)	0 (0)	“It was good to be made aware of how I was feeling at that moment.” [PT5]
What makes the surgery worthwhile?	13 (76)	4 (24)	0 (0)	“A really good question. A question I would have liked to have asked myself two years ago, because this is what it is really about, in the end. Why to choose for surgery or not.” [PT12]
Formulating positive statements	12 (71)	5 (29)	0 (0)	“It made me start a conversation with my spouse about my recovery process.” [PT6]
Mindful enjoying	11 (65)	3 (18)	3 (18)	“Walking is something you do without thinking about it. And before surgery it was terrible, walking. So becoming aware of the fact that walking is possible without pain...It’s good to become aware of these things.” [PT6]
What do you desire to do (again) after surgery?	11 (65)	4 (24)	2 (12)	“It made me melancholic or sad, because it were things that I had not been able to do for a very long time.” [PT7]
3 positive things	9 (53)	5 (29)	3 (18)	“Yes, I absolutely loved it. I was busy in the kitchen, cooking. I was able to do that all by myself again. It was really a great day.” [PT11]
Wish question	8 (47)	7 (41)	2 (12)	“It got me out of the moment when I was in pain and helped me to see what made it worthwhile.” [PT7]
Mindfulness exercises	6 (35)	5 (29)	0 (0)	“It has really helped me, especially in the beginning. Further along during recovery I didn’t need it anymore.” [PT14]
Uploading a valuable picture	6 (35)	5 (29)	6 (35)	“I could upload a picture of my grandchildren, but I don’t see the use or need for such an exercise.” [PT5]
Video of how pain works	4 (24)	5 (29)	8 (47)	“That really was an eye-opener for me. It all fell into place.” [PT12]
Write a letter to yourself	2 (12)	15 (88)	0 (0)	“I really did not see the use of this exercise.” [PT9]

The potential relaxing effect of the mindfulness exercises was not experienced by 12% (2/17) of the participants (PT3 and PT9) even though they did do the exercises. Other participants (4/17, 24%) stated that the exercises were not for them as they already knew the techniques (eg, from yoga) but suggested that they might benefit other patients. The least appreciated exercise was writing a letter to themselves to support themselves in difficult times during recovery. Exercises focusing on values and positive statements were the most appreciated by the participants. Table 3 provides a more detailed overview.

#### Suggestions for Improvement

##### Overview

The participants also gave some suggestions for improvement. For instance, PT1, PT2, PT6, and PT15 stated that the information in the intervention was sometimes too elaborate and could have been more concise. Some suggestions about the usability of the intervention were mentioned (eg, on where to position the menu of the intervention or to enlarge the font size for older participants). Several participants would have liked the intervention to have less psychological content (eg, PT4, PT12, and PT15) or at least have the option to influence the amount of these exercises or content while using the intervention (eg, PT17). Other points of interest were the need for more specific physical guidelines (eg, PT11), more physical (therapy) exercises (PT18), and information about returning to work (PT17).

##### Engagement With the Intervention

Engagement with the intervention is described through the topics *usage*, *reasons for not using*, and *number of modules*, as discussed in the interviews, together with scores on the TWEETS and log data.

In the interviews, some participants (3/17, 18%) mentioned that they mainly used the intervention *before* surgery, and PT1 even used it almost daily before surgery. Other participants primarily used the intervention *after* surgery (eg, “almost daily to check the physical guidelines” [PT2 and PT11]), especially the first few weeks after surgery, when they were more restricted in movements and were in bed for most of the day (eg, PT9, PT12, PT14, and PT17). These participants stated that, as their recovery—and, therefore, their mobility—progressed, their use of the intervention declined:

I noticed that the more I recovered, the less I used the app.

PT9

Other participants used the intervention *both before and after surgery* (eg, PT18 and PT3):

I must have read the entire app at least 10 times, I only missed one weekly module because I got COVID.

PT3

Interestingly, all participants stated that the fact that they received a new module every week helped them use the intervention more often, as did viewing the other content of the intervention while filling out the weekly module.

Reasons for not using the intervention (more) were the lack of automatic notifications or reminders (eg, PT16), feeling no pain postoperatively, feeling that pain management was the main focus of the intervention (PT11), or lack of interest in the (large amount of) psychological content (eg, PT9, PT11, PT16, and PT17).

Most participants (12/17, 71%) felt that the number of modules was correct. The other 29% (5/17) of the participants stated that fewer modules would have sufficed, especially in the later weeks after surgery.

The mean total score of all participants in the intervention group on the TWEETS was 2.6 out of a possible 4 (SD 0.7), which is more than the average answering option of the scale (65% of the maximum score). The highest-scoring items on the TWEETS were “*Strength Back* is easy to use” and “*Strength Back* helps me to get more insight into my preparation before surgery and recovery after surgery” with an average score of 2.9 and 3.1, respectively. The lowest-scoring items on the TWEETS were “*Strength Back* is part of my daily routine” with an average score of 1.9 and “*Strength Back* fits me as a person” with an average score of 2.1.

A total of 15 weekly modules were offered to the patients undergoing spinal fusion (6/17, 35%). Log data of these patients showed that the average number of weekly modules completed was 10.2 out of 15 (SD 3.9; Table 4). The least completed weekly modules were POST-6, POST-7, and POST-12 (2/6, 33% in all cases). All patients undergoing spinal fusion completed the weekly POST-2 and POST-3 modules.

**Table 4:** Overview of completed weekly modules per participant—patients undergoing spinal fusion (N=6).

Participant number	PRE				POST													Total, n (%)^a^
	1	2	3	1		2	3	4	5	6	7	8	9	10	11	12		
PT3				✓		✓	✓	✓	✓	✓	✓	✓	✓	✓	✓		11 (73)	
PT5	✓	✓	✓	✓		✓	✓	✓	✓	✓	✓	✓	✓	✓	✓	✓	15 (100)	
PT8	✓					✓	✓		✓				✓				5 (33)	
PT9	✓	✓	✓	✓		✓	✓	✓	✓			✓	✓	✓		✓	12 (80)	
PT12	✓	✓	✓	✓		✓	✓	✓	✓			✓	✓	✓	✓		12 (80)	
PT17	✓	✓	✓	✓		✓	✓										6 (40)	
Total, n (%)^b^	5 (83)	4 (67)	4 (67)	5 (83)		6 (100)	6 (100)	4 (67)	5 (83)	2 (33)	2 (33)	4 (67)	5 (83)	4 (67)	3 (50)	2 (33)	N/A^c^	

^a^Total number of completed modules; maximum of 15 modules.

^b^Total number of participant completing a specific module; maximum of 6 participants.

^c^N/A: not applicable.

Several participants kept using the intervention until the last weekly module. The least completed weekly modules for patients undergoing decompression surgery were the PRE-module 3 (5/11, 45%) and POST-module 5 (4/11, 36%), whereas the PRE-module 1 was completed by almost all participants (10/11, 91%; Table 5).

**Table 5:** Overview of completed weekly modules per participant—patients undergoing decompression surgery (N=11).

Participant number	PRE				POST							Total completed, n (%)^a^
	1	2	3	1		2	3	4	5	6		
PT1	✓	✓	✓	✓		✓	✓	✓	✓	✓	9 (100)	
PT2	✓	✓		✓				✓			4 (44)	
PT4	✓	✓		✓		✓	✓	✓	✓	✓	8 (89)	
PT6	✓			✓				✓			3 (33)	
PT7	✓	✓	✓	✓		✓	✓	✓			7 (78)	
PT10		✓	✓			✓	✓		✓	✓	6 (67)	
PT11	✓	✓		✓		✓	✓	✓	✓	✓	8 (89)	
PT13	✓			✓		✓				✓	4 (44)	
PT14	✓	✓	✓	✓		✓	✓	✓		✓	8 (89)	
PT15	✓	✓									2 (22)	
PT16	✓	✓	✓							✓	4 (44)	
Total respondents, n (%)^b^	10 (91)	9 (82)	5 (45)	8 (73)		7 (64)	6 (55)	7 (64)	4 (36)	7 (64)	N/A^c^	

^a^Total number of completed modules; maximum of 9 modules.

^b^Total number of participant completing a specific module; maximum of 11 participants.

^c^N/A: not applicable.

## Discussion

### Principal Findings

The objective of this study was 3-fold: to explore the potential benefits for patients undergoing spinal surgery of the digital ACT and PPI *Strength Back* (RQ 1), explore the feasibility of a future RCT in terms of recruitment and dropout (RQ 2), and assess the *acceptability* of *Strength Back* by patients undergoing spinal surgery (RQ 3).

The focus of this study was to explore the potential benefits of the intervention and not to determine its effectiveness. The latter should be the focus of a future RCT including more participants and allowing for more robust statements. Nonetheless, this study does show that *Strength Back* seems promising as pain intensity decreased more in the intervention group than in the control group and emotional well-being as well as overall well-being improved in the intervention group but not in the control group (RQ 1). This is in line with research in patients with chronic pain showing beneficial effects of ACT and PPIs compared with a control group on pain intensity and emotional functioning [65,66], in the treatment of chronic pain [30-35], in improving affect and functional ability after knee surgery [21], and in quicker cessation of pain and opioid use in veterans after orthopedic surgery [36].

Contrary to our expectations, no differences between the groups were observed in improvement in pain interference and psychological flexibility. Possibly, the effect of the ACT and PPI content shows through in the significantly larger decline in pain intensity in the intervention group. It is reasonable to suppose that a decline in pain intensity also helps prevent pain interference with daily activities and improve psychological flexibility. However, the sample size may have been too small, or the effect of surgery may have been so massive that these effects did not appear in this study. Moreover, in the intervention group as well as the control group, valued living and psychological and social well-being were almost the same at the pre- and posttest time points. The amount of ACT and PPI content may have been too small to influence valued living or psychological and social well-being. It is also possible that these effects only occur in the longer term. Living more accordingly to one’s values, resulting in enhanced psychological and social well-being, may only start later in the recovery process and not immediately after surgery.

In terms of recruitment and dropout rates, a future RCT seems feasible (RQ 2). A large proportion of the approached patients wanted to participate in the study, and once they started with the intervention, almost all participants used it until the posttest assessment and final interview. The willingness to use a digital health intervention seemed quite high in our study compared with in previous studies [67,68]. It seems that, once participants started the intervention in this study, they saw its value and kept using it over a longer period. In line with other research [69], patients stated that receiving notifications improved their use of the intervention. The dropout numbers found in this study were low and correspond to those of a recent RCT on PP exercises for patients with chronic pain [66]. However, to recruit a larger number of participants for an RCT, multiple hospitals or orthopedic centers should be included as there are a limited number of potential participants in a single facility. Interestingly, almost all the patients who were approached and did not want to participate in our study were female. Perhaps this group needs more attention during recruitment to explore their reasons for not wanting to participate.

The vast majority of participants in the intervention group were positive about the digital health intervention and would recommend it to future patients (RQ 3). The scores on the TWEETS indicated a moderate to above-moderate engagement with the intervention, which was in line with log data showing that participants completed on average approximately 75% of the available weekly modules. The information modules containing videos of the surgery room and nursing ward, practical tips from previous patients, physical activity guidelines, and pain medication were the most appreciated by participants. The PP and ACT content evoked the strongest opinions in the participants, with a small minority indicating that they preferred no psychological content at all, whereas most of the participants saw added value in a number of specific exercises. Although the development of the intervention was based on a participatory design process and most participants appreciated the content, others felt that the focus was too much on mental health, whereas they experienced their issues as physical. At the same time, to increase the effectiveness of the intervention on the ACT- and PP-related outcomes, even more psychoeducation and exercises might be needed. In addition, the current version of *Strength Back* focuses on certain elements of ACT, whereas it may be necessary to address all ACT processes to achieve optimal benefits for patients undergoing spinal surgery. Indeed, Carr et al [29] found in their meta-analysis of PPIs that interventions were more effective when they contained multiple PPIs, were of longer duration, and contained more sessions. This poses a dilemma for the further development of this intervention. Information on the psychological content of the intervention before inclusion and tailoring the content and dose of the intervention to individual patients might further improve acceptance of the PP exercises, as suggested by previous research [70-72]. In a future version of *Strength Back*, patients could be introduced with a few of the most appreciated ACT and PP exercises and from there on be given the possibility to determine the amount of ACT and PP content themselves. This might reduce the potential effect on some patients who opt for a lower amount of ACT and PP content but might increase overall adherence to and acceptance of the intervention.

Our results question what the most appropriate primary outcome is for a subsequent RCT. As the intervention is based on ACT and PP, it makes sense to choose a measure corresponding to this content, such as well-being or pain interference. However, our feasibility study shows that the intervention and control groups particularly differed in improvement in pain intensity, which is an outcome that is important to patients but not directly targeted within the intervention. Perhaps targeting both pain intensity and increasing well-being might be the best option for sustainable resilience in patients undergoing spinal surgery. This is in line with previous research proposing a balanced, complaint and strength–oriented approach to reach sustainable mental health [73].

Second, attention should be paid to what the appropriate process measurements in a subsequent RCT would have to be. On the basis of the content and assumed working mechanism of this intervention, we propose *psychological flexibility* as a process measure in a future RCT. Nonetheless, we would not advise using the PIPS to measure this concept as we found low reliability in our study. Other studies have found similar issues with this scale and have recommended only interpreting it as a whole [74] or only using the avoidance subscale [75]. In addition, the PIPS measures inflexibility and not flexibility, which does not fit as well with the focus of ACT and PP. Perhaps the Personalized Psychological Flexibility Index [76] is a suitable alternative measure of psychological flexibility for a future RCT. In addition, we propose *pain interference* as a process measure in a future RCT, measured using the MPI subscale of pain interference. In addition, the ELS might be used in a future RCT to measure engaged living, including valued living.

The final discussion point relates to the length and timing of the intervention. The current intervention starts before surgery and lasts 6 to 12 weeks after surgery (for decompression and spinal fusion surgery, respectively). This was regarded positively by most participants, and from both the interviews and log data, we learned that most participants were already active in the intervention before surgery. This focus on pre- and postoperative timing is supported by a recent systematic review on perioperative psychological interventions that found that interventions (also) delivered after surgery tended to be more effective for postsurgical pain and disability than interventions delivered (only) before surgery [77]. It seems that, while waiting for surgery, patients are quite eager to learn more about the surgery and rehabilitation afterward. An intervention such as *Strength Back* can take advantage of these “teachable moments” [39] by providing content that participants may not have found or chosen themselves but that is known to help them in the recovery process. The duration of our intervention is in line with a recent review on perioperative psychological interventions for patients undergoing spinal fusion showing a reduction in pain and disability starting from immediately after surgery up to 3 months [78].

### Strengths of This Study

The high recruitment and relatively low dropout rates were a strength of this study. In addition, qualitative data were collected through individual interviews with almost all the users of the intervention. This yielded crucial information on the value of the intervention and the reasons behind participants’ use of the intervention. Using multiple methods combining qualitative data with log data and quantitative data retrieved through the questionnaires provided a complete picture of the value of the intervention. Therefore, this research design is close to a convergent parallel mixed methods design [79]. Another strength of this study is the underlying theoretical framework of the intervention. As this is lacking for many digital health interventions, there are calls for further research to enhance the scope and use of this technology [80,81]. The design of digital interventions should be anchored in behavior change theories to optimally engage patients in the intervention and behavior change [82,83]. In a review of 85 studies using the Behavior Change Support Systems by Oinas-Kukkonen and Harjumaa [82], less than half referred to theories of behavior change, but those that did were uniformly successful [84]. Clearly, a sound theoretical base for digital health interventions is warranted for optimal behavior change and effectiveness.

### Limitations of This Study

As all participants underwent surgery, which is an intervention by itself, we wanted to see in this study what the digital health intervention might add to the effect of the surgery. An exploratory design regarding outcome measures was used to see which potential benefits could be achieved for patients undergoing spinal surgery. Owing to this exploratory design and the small sample size, more and larger effects of the intervention were not expected to be found in this study. In addition, no correction for multiple testing was performed in this study, and thus, conclusions can only be drawn with caution. Therefore, the preliminary findings of this study need to be replicated in a future larger study.

This study used a historic control group. A future RCT should use a design in which the intervention group and the control group run parallel in time and participants are assigned to them randomly.

In this study, log data were gathered on modules in which participants answered questions. For a future study, we would recommend registering every log-in and activity of users instead of only activities in which participants provide answers. We now see certain patterns in the use of participants, but more data are needed to further investigate use of and engagement with the intervention.

Previous hospital experience, preexisting comorbidities, patient technology readiness, health literacy, and ward factors such as staffing were not measured in this study. As these factors may influence how prepared patients are and what their capability is to engage with the intervention, they should be considered in a future RCT.

### Conclusions

This study shows that combining ACT and PP in a digital health intervention is promising for patients undergoing spinal surgery as the content was accepted by most of the participants and (larger) improvements in pain intensity and well-being were found in the intervention group. A digital intervention for patients undergoing (spinal) surgery can use teachable moments, when patients are open to learning more about the surgery and rehabilitation afterward, to also provide patients with content that they may not have found or chosen themselves but that is known to help them in the recovery process.

## Appendix

Multimedia Appendix 1 Content of the Strength Back digital health intervention for patients undergoing spinal surgery.

Multimedia Appendix 2 Overview of positive psychology and acceptance and commitment therapy exercises in the weekly modules of the Strength Back digital health intervention for patients undergoing spinal surgery.

Multimedia Appendix 3 Overview of the timing of the positive psychology and acceptance and commitment therapy exercises in the weekly modules of the Strength Back digital health intervention for patients undergoing spinal surgery.

## Abbreviations

ACT: acceptance and commitment therapy
ELS: Engaged Living Scale
HADS: Hospital Anxiety and Depression Scale
MPI: Multidimensional Pain Inventory
NRS: Numeric Pain Rating Scale
PIPS: Psychological Inflexibility in Pain Scale
PP: positive psychology
PPI: positive psychology intervention
RCT: randomized controlled trial
RQ: research question
TWEETS: Twente Engagement With eHealth Technologies Scale

## Data Availability

The data sets analyzed during this study are not publicly available because of the exploratory and mixed methods nature of the study, but they are available from the corresponding author upon reasonable request.
