# Supplementary material for: Benefits, Recruitment, Dropout, and Acceptability of the Strength Back Digital Health Intervention for Patients Undergoing Spinal Surgery: Nonrandomized, Qualitative, and Quantitative Pilot Feasibility Study
Source: JMIR Form Res. 2024 Feb 7;8:e54600. doi: 10.2196/54600 (PMC10882475; doi:10.2196/54600)
Supplement: Multimedia Appendix 1 [file formative_v8i1e54600_app1.docx]

Appendix 1. Content of the digital health intervention Strength Back for spinal surgery patients.

| Module title | Module content |
| --- | --- |
| Introduction | Information about the function and content of the intervention |
| Information on spinal condition | Information about stenos or spondylolisthesis |
| Information about the surgery | Information about surgical procedure |
| Physical guidelines | Physical guidelines per week on what to do and not to do after surgery |
| Contact the hospital | List of complications when to contact the hospital  Contact details of the hospital |
| Videos | Video of virtual tour through the nursing ward  Video of virtual tour through the surgery room |
| Pain medication | Information about types of pain medication, their function, side effects and tapering of medication |
| Mindfulness exercises | Mindful breathing and body scan |
| How does pain work? | Pain education including animation video explaining pain mechanisms |
| Physical therapy | Information about physical therapy before and after surgery  Physical criteria for discharge from the hospital |
| Practical tips | Tips from previous spinal surgery patients |
| Quotes from others | Quotes from previous spinal surgery patients |
| Recovery | Text and quotes on the ups and downs during recovery |
