# Supplementary material for: Benefits, Recruitment, Dropout, and Acceptability of the Strength Back Digital Health Intervention for Patients Undergoing Spinal Surgery: Nonrandomized, Qualitative, and Quantitative Pilot Feasibility Study
Source: JMIR Form Res. 2024 Feb 7;8:e54600. doi: 10.2196/54600 (PMC10882475; doi:10.2196/54600)
Supplement: Multimedia Appendix 2 [file formative_v8i1e54600_app2.docx]

Appendix 2.

| Name of the exercise | Content of the exercise | Positive Psychology (PP) and/or ACT |
| --- | --- | --- |
| Mindfulness exercises | Mindful breathing and body scan | ACT (contact with present moment) |
| Wish question | What if you could make a wish for your health? What would this change? And how can you already make a step in that direction today? | ACT (values and committed action) |
| What makes the surgery worthwhile? | What are value-based activities to do (again, after surgery) or keep doing? | ACT (values and committed action) |
| A letter to yourself | Write a letter to encourage yourself in hard times, e.g. when recovery is tough | PP |
| Positive statements | Formulate statements to encourage yourself in hard times, e.g. when recovery is tough | PP |
| Valuable image | Which picture (on your mobile phone) shows what you find important and valuable, how can you use this during recovery (e.g. as a goal)? | ACT (values and committed action) |
| Three positive things | Which three things made you grateful today and what was your own part in this? | PP |
| Mindful enjoying | Which activities have you undertaken today that often go without attention, but which, today, you have consciously engaged in and enjoyed? E.g. cooking, taking a shower or walking? | PP |

Overview of positive psychology and ACT exercises in the weekly modules of the digital health intervention Strength Back for spinal surgery patients.
