# Supplementary material for: Benefits, Recruitment, Dropout, and Acceptability of the Strength Back Digital Health Intervention for Patients Undergoing Spinal Surgery: Nonrandomized, Qualitative, and Quantitative Pilot Feasibility Study
Source: JMIR Form Res. 2024 Feb 7;8:e54600. doi: 10.2196/54600 (PMC10882475; doi:10.2196/54600)
Supplement: Multimedia Appendix 3 [file formative_v8i1e54600_app3.docx]

Appendix 3. Overview of timing of positive psychology and ACT exercises in the weekly modules of the digital health intervention Strength Back for spinal surgery patients.

| Name of exercise | Timing of exercise |
| --- | --- |
| Mindfulness exercise* | PRE1** + POST2/POST9*** |
| Wish question | PRE1 + POST2/POST3 |
| What makes the surgery worthwhile? | PRE2 + PRE3 |
| A letter to yourself | PRE2 + POST2/POST8 |
| Positive statements | PRE2 + POST4/POST5 |
| Valuable image | POST3/POST4 + POST10 |
| Three positive things | POST5/POST7 |
| Conscious enjoyment | POST5/POST6 + POST11 |

Note: *Mindfulness exercises are also constantly available in a separate module; **PRE=weekly module before surgery, POST=weekly module after surgery; ***Timing for decompression/spinal fusion version
